# Bioinformatics profiling integrating a four immune-related long non-coding RNAs signature as a prognostic model for papillary renal cell carcinoma

**DOI:** 10.18632/aging.103580

**Published:** 2020-07-27

**Authors:** Yu Liu, Xin Gou, Zongjie Wei, Haitao Yu, Xiang Zhou, Xinyuan Li

**Affiliations:** 1 Department of Urology, The First Affiliated Hospital, Chongqing Medical University, Chongqing, China; 2 Chongqing Key Laboratory of Molecular Oncology and Epigenetics, Chongqing, China; 3 Department of Urology, Chongqing Traditional Chinese Medicine Hospital, Chongqing, China

**Keywords:** papillary renal cell carcinoma, immune, long non-coding RNAs, risk score, prognosis

## Abstract

Background: Papillary renal cell carcinoma (pRCC) was the 2^nd^ most common subtype, accounting for approximately 15% incidence of renal cell carcinoma (RCC). Immune related long non-coding RNAs (IR-lncRs) plentiful in immune cells and immune microenvironment (IME) are potential in evaluating prognosis and assessing the effects of immunotherapy. A completed and meaningful IR-lncRs analysis based on abundant pRCC gene samples from The Cancer Genome Atlas (TCGA) will provide insight in this field.

Results: 17 IR-lncRs were selected by Pearson correlation analysis of immune score and the lncRNA expression level, and 5 sIRlncRs were significantly correlated with the OS of pRCC patients. 4 sIRlncRs (AP001267.3, AC026471.3, SNHG16 and ADAMTS9-AS1) with the most remarkable prognostic values were identified to establish the IRRS model and the OS of the low-risk group was longer than that in the high-risk group. The IRRS was certified as an independent prognosis factor and correlated with the OS. The high-risk group and low-risk group showed significantly different distributions and immune status through PCA and GSEA. In addition, we further found the expression levels of SNHG16 was remarkably enhanced in female patients with more advanced T-stages, but ADAMTS9-AS1 showed the opposite results.

Conclusion: The IRRS model based on the identified 4 sIRlncRs showed the significant values on forecasting prognoses of pRCC patients, with the longer OS in the low-risk group.

Methods: We integrated the expression profiles of LncRNA and overall survival (OS) in the 322 pRCC patients based on the TCGA dataset. The immune scores calculated on account of the expression level of immune-related genes were used to verify the most relevant IR-lncRs. Survival-related IR-lncRs (sIRlncRs) were estimated by COX regression analysis in pRCC patients. The high-risk group and low-risk group were identified by the median immune-related risk score (IRRS) model established by the screened sIRlncRs. Functional annotation was displayed by gene set enrichment analysis (GSEA) and principal component analysis (PCA), and the immune composition and purity of the tumor were evaluated through microenvironment cell count records. The expression levels of sIRlncRs of pRCC samples were verified by real-time quantitative PCR.

## INTRODUCTION

Renal cell carcinoma (RCC) is one of the worldwide commonest cancers, with the 9^th^ highest incidence among all carcinomas and the most common pathological pattern in all kidney malignant tumors [1]. In 2004, World Health Organization (WHO) identified 11 histologic types of RCC, of which papillary RCC (pRCC), following clear cell RCC (ccRCC), was the 2^nd^ most common subtype, accounting for approximately 15% incidence of RCC [2, 3]. Characterized by the heterogeneous multifocal or isolated tumor, pRCC also showed indolent or aggressive the two distinguish behavioral features [4]. In 2016, the WHO revised the new classification of renal tumors, of which pRCC was further classified into types I and II in greater detail [5, 6]. Given the unique clinicopathological features of various subtypes, series of researchers began paying attentions to discovery of pRCC.

With the deeper understanding of the crucial roles of immune and stromal cells on tumor biological progress, a growing body of researchers were motivated to discover immunotherapy of pRCC, and increasing immunotherapy drugs obtained rewarding treatment effects [7–10]. However, the limited response rate and the emergence of resistance also occurred in another parts of patients [11]. Therefore, researchers started focusing subsequent studies on identifying more accurate and sensitive biomarkers to distinguish patients probably with the satisfied therapy response rate and prognosis, so as to achieve greater benefits on therapeutic efficiency and survival [12].

With the increasingly clear cognition of the crucial role of genetics and epigenetics on tumor pathological features, biological behaviors, therapy strategies and prognosis, more pRCC-related immune genes and genetic modification approaches were identified to be involved in the diagnosis process and prognosis prediction [13, 14] Long non-coding RNAs (lncRNAs) are a kind of transcripts lacking the potential of coding proteins, but showed the pivotal position, such as antigen exposure, recognition and immune infiltration [15]. Thus, the potential of immune-related lncRNAs (IR-lncRs) on forecasting tumor progression and prognosis are drawing increasing attentions. Xiao Y. detected the prognostic effect of lncR-H19 in glioma [16]. Chen S. revealed that the expression level of lncR-PVT1 was significantly associated with the overall survival (OS) of patients with osteosarcoma [17].

However, the potential of certain genetic markers in immune microenviroment (IME) especially for iIR-lncRs on prognosis forecast have yet to be adequately elucidated [18, 19]. Therefore, identifying some novel and sensitive genetic biomarkers which are potential in predicting progression and prognosis of pRCC might be helpful to provide more personalized guideline and appropriate therapeutic strategy.

To give an insight into the clinical potency of IME and IR-lncRs on prognosis evaluation of pRCC, we extracted a class of IR-lncRs in IME predicting poor prognosis in pRCC patients, together with their clinicopathologic signatures, we further calculated their correlations with OS. The results establish a more personalized precision predicting model of pRCC, and provide the guiding light for making clinical decision.

## RESULTS

### Acquisition of IR-lncRs

Transcriptome data and clinical data of patients with pRCC were fetched from TCGA database. Next, transcriptome data was processed to convert the data ensembl ID into gene names. Following that, transcriptome data were divided into lncRNA and mRNA. From the Immune system process M13664 and Immune response M19817 of Molecular Signatures Database, we identified 331 pRCC IRGs, of which 17 lncRNAs were validated to be the IR-lncRs by correlation analysis.

### The relevance of IR-lncRs and prognosis

**Figure 1:**
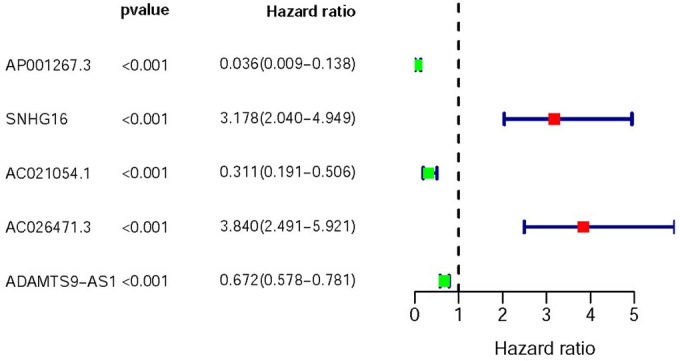
**Survival-related values of sIRlncRs.** Forest plot of hazard ratios showing the survival-related values of sIRlncRs (AP001267.3, SNHG16, AC021051.1, AC026471.3 and ADAMTS9-AS1). Red parts represent upregulated sIRlncRs, and green parts represent downregulated sIRlncRs.

Based on COX Regression model, we then identified 5 IR-lncRs which were associated with prognosis (sIRlncRs), such as AP001267.3, SNHG16, AC021054.1, AC026471.3 and ADAMTS9-AS1. The relationships between these sIRlncRs and prognosis were clearly illustrated in the forest map (Figure 1).

### Clinicopathologic characteristics of the high-risk group and the low-risk group

**Figure 2:**
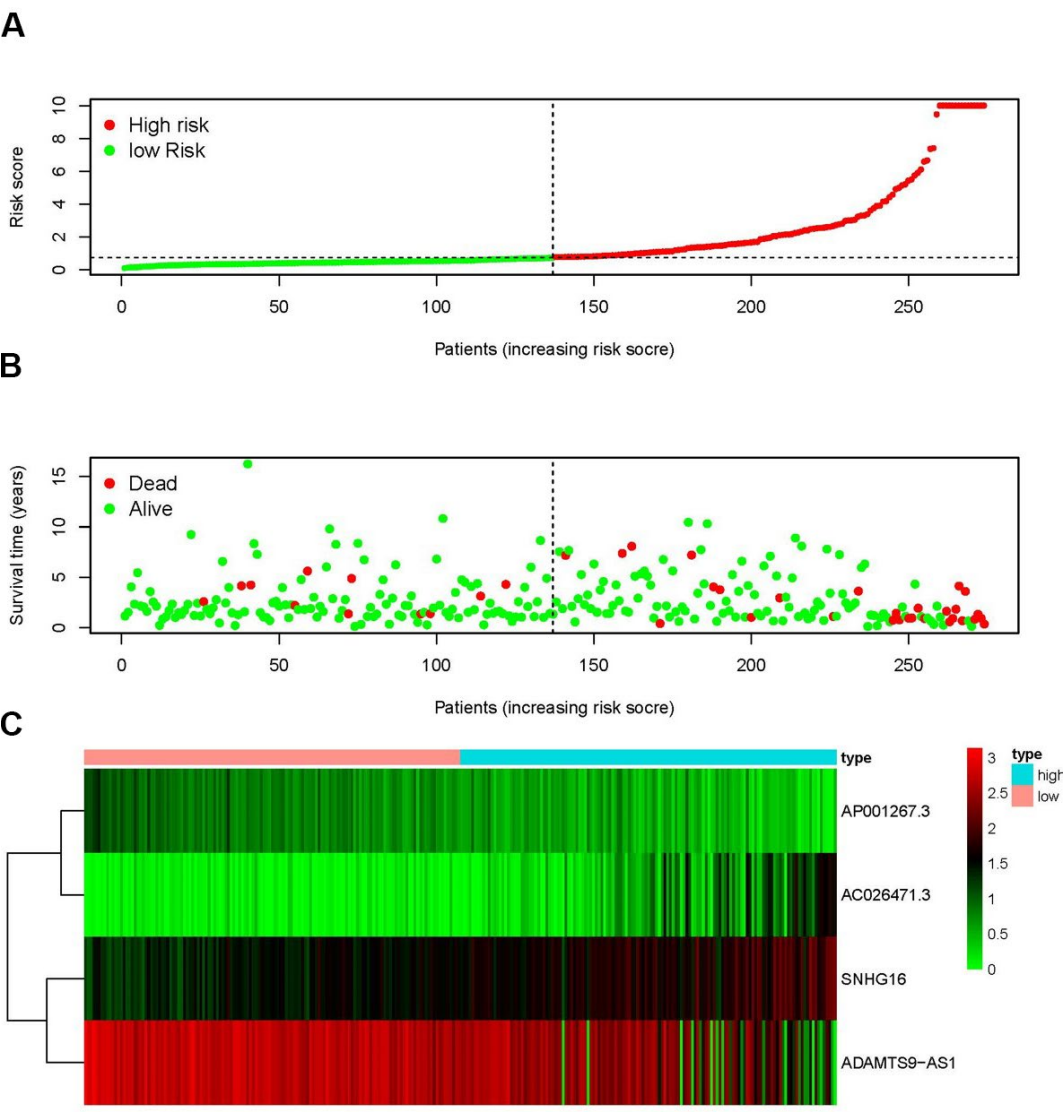
**IRRS was established according to sIRlncRs.** Distribution of risk score in the high-risk group and the low-risk group (**A**). Survival status between the high-risk group and the low-risk group (**B**). The heatmap of expression profile of contained sIRlncRs (**C**). In the heatmap, red parts represent up-regulation, green parts represent down-regulation, and black parts represent sIRlncRs without differential expression.

**Figure 3:**
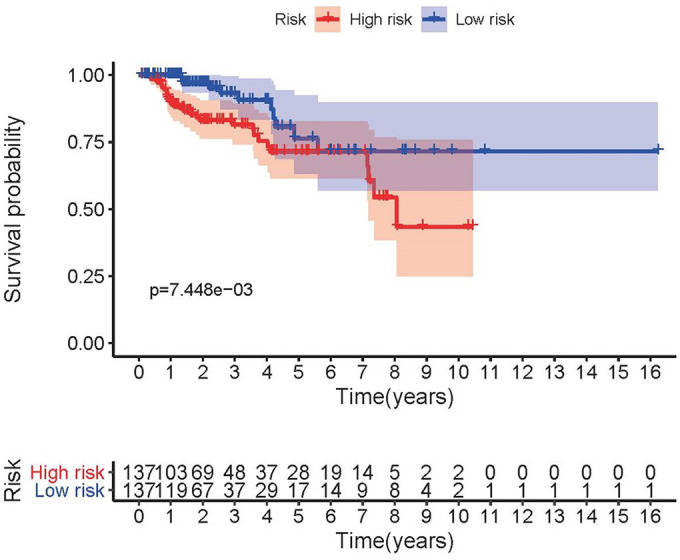
**Survival curve of pRCC patients.** Kaplan-Meier survival curve of OS among pRCC patients from the low-risk group and the high-risk group. The high-risk group showed the poorer prognosis.

The top 4 sIRlncRs (AP001267.3, AC026471.3, SNHG16 and ADAMTS9-AS1) among the 5 sIRlncRs were included to establish the risk evaluating model, by which the pRCC samples were divided into the high-risk group and the low-risk group based on the intermediate risk score (Figure 2A). The mortality rate constantly increased with the higher risk score (Figure 2B). And with the increase of risk score, the expression levels of AC026471.3 and SNHG16 were elevated, while AP001267.3 and ADAMTS9-AS1 expressed decreasingly (Figure 2C). The survival of the high-risk group was significantly shorter than that of the low-risk group (Figure 3).

### The clinical application of the IRRS and the relationships between the IRRS and clinicopathologic indicators

**Figure 4:**
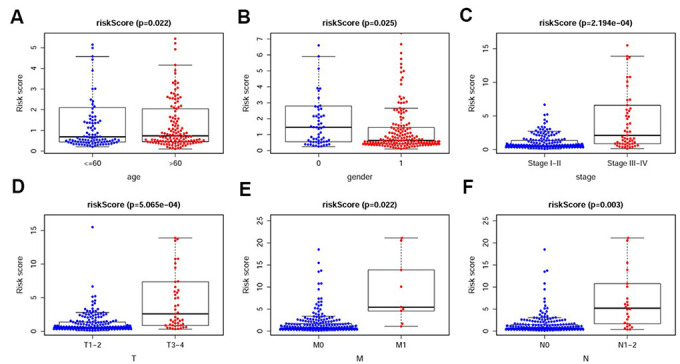
**The relationships between the IRRS and different clinicopathological features.** Relationships between the IRRS and age (**A**), gender (**B**), tumor stage (**C**), T-stage (**D**), M-stage (**E**) and N-stage (**F**). (0=Female patients; 1=Male patients).

To investigate the relevance of the sIRlncRs and clinicopathological features of pRCC, we analyzed the correlation between the risk score and the clinical and demographic characteristics, such as age, gender, stage, T-stage, N-stage and M-stage. Under the IRRS, the scores of older patients (Figure 4A), female patients (Figure 4B), patients with advanced stage (Figure 4C), advanced T-stage (Figure 4D), advanced M-stage (Figure 4E) and advanced N-stage (Figure 4F) were significantly increased. The above results elucidate some clinical and demographic characteristics that are sensitive to the IRRS and further corroborate the clinicopathological application value of the model.

**Figure 5:**
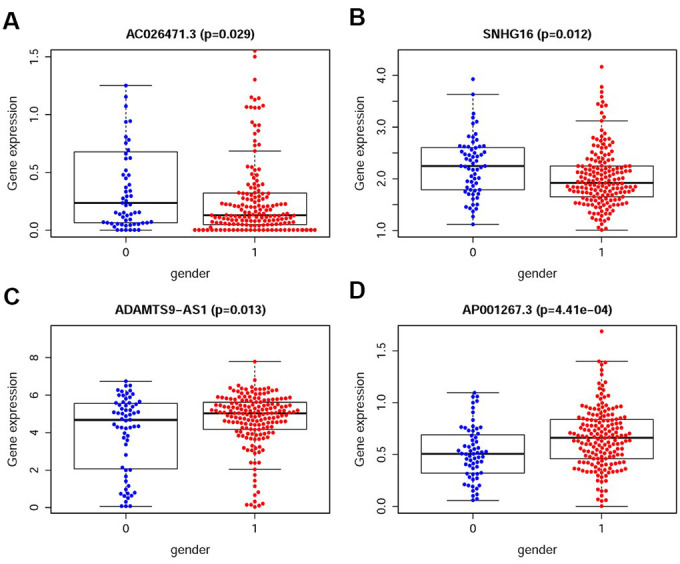
**The relationships between the sIRlncRs and gender.** The expression of AC026471.3 (**A**) and SNHG16 (**B**) were lower in male patients. The expression of ADAMTS9-AS1 (**C**) and AP001267.3 (**D**) were lower in female patients. (0=Female patients; 1=Male patients).

We also analyzed the relationships between the compositions of the IRRS and the aforementioned tumor characteristics which all illustrated in Table 1. The expression levels of SNHG16 and AC026471.3 were higher in female patients (Figure 5A–5B). However, the expression levels of AP001267.3 and ADAMTS9-AS1 were lower in female patients (Figure 5C–5D).

**Table 1:** The relationships between the compositions of IRRS and the clinical characteristics.

**Genes**	**t(*P*)**					
	**Age (≥60/<60)**	**Gender (male/female)**	**Stage (III-IV/I-II)**	**T-stage (T3-4/T1-2)**	**M-stage (M1/M0)**	**N-stage (N1-3/N0)**
**AP001267.3**	-1.636(0.103)	-3.615(4.41e-04)	5.646(1.891e-07)	5.586(3.387e-07)	4.345(0.002)	7.185(2.505e-08)
**SNHG16**	1.38(0.169)	2.567(0.012)	-5.338(1.036e-06)	-5.711(3.742e-07)	-3.601(0.006)	-4.065(3.414e-04)
**AC026471.3**	0.788(0.432)	2.228(0.029)	-4.274(5.91e-05)	-4.366(5.181e-05)	-3.414(0.009)	-3.883(6.072e-04)
**ADAMTS9-AS1**	-0.958(0.339)	-2.551(0.013)	5.065(2.521e-06)	4.88(6.979e-06)	4.358(0.002)	4.979(2.601e-05)
**Risk score**	2.324(0.022)	2.3(0.025)	-3.946(2.194e-04)	-3.724(5.065e-04)	-2.823(0.022)	-3.341(0.003)

Note: t: t value of student's t test; *P*: *P-*value of student's t test.

**Figure 6:**
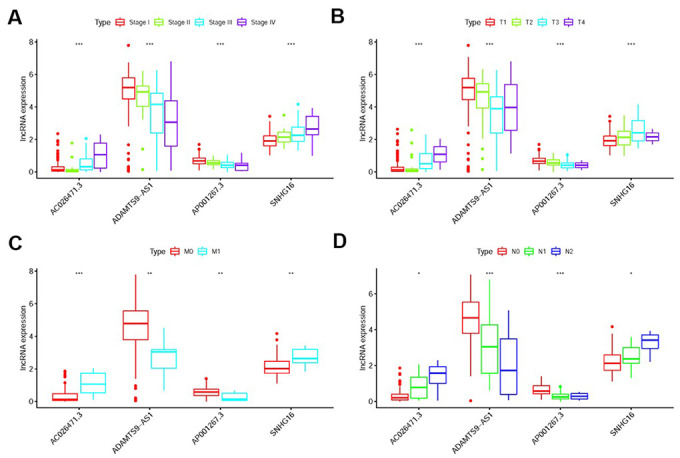
**The relationships between the sIRlncRs and clinicopathological features.** Relationships between sIRlncRs (AP001267.3, AC026471.3, SNHG16 and ADAMTS9-AS1) and clinicopathological features. The expression of AC026471.3 and SNHG16 were gradually increased in the more advanced stage (**A**), T-stage (**B**), M-stage (**C**) and N-stage (**D**), while the expression levels of AP001267.3 and ADAMTS9-AS1 were gradually decreased. (*P<0.05; **P<0.01; ***P <0.001).

We further found the expression levels of AP001267.3 and ADAMTS9-AS1 were gradually decreased in the more advanced stage, T-stage, M-stage and N-stage, while the expression levels of AC026471.3 and SNHG16 were enhanced (Figure 6A–6D). Detection of the roles of different sIRlncRs in indicating tumor characteristics provides insight into the further discovery of biomarkers.

**Figure 7:**
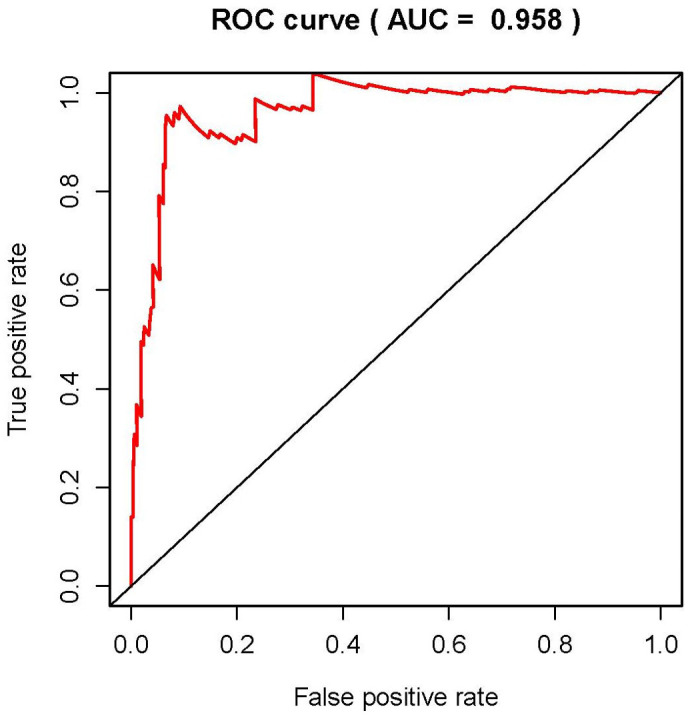
**Receiver operating characteristic (ROC) curve.** ROC curves indicate the prognostic value of independent prognostic factors. The AUC is 0.958.

We then conducted the independent risk analysis, the results showed age, stage, N-stage, M-stage and risk score were significantly correlated with OS in univariate analysis (P<0.05). But in the multivariate analysis, M-stage and risk score showed more remarkable correlations with OS (Table 2). The ROC curve represented the accuracy of the model. The AUC of risk score was 0.958 (Figure 7). These results suggested the risk score was an independent prognostic factor.

**Table 2:** Univariate and multivariate analysis of pRCC.

**Variables**	**Univariate analysis**				**Multivariate analysis**			
	**HR**	**HR 95% low**	**HR 95% high**	***P* value**	**HR**	**HR 95% low**	**HR 95% high**	***P* value**
**Age**	0.959064	0.920145	0.999629	0.047984	0.985610	0.917089	1.059249	0.693388
**Gender**	1.451893	0.404361	5.213146	0.567527	0.458258	0.070090	2.996123	0.415339
**Stage**	2.350466	1.286794	4.293375	0.005431	1.837786	0.254958	13.24707	0.545934
**T-stage**	1.718677	0.920973	3.207317	0.088879	0.656824	0.101067	4.268619	0.659808
**M-stage**	70.58455	8.169804	609.8283	0.000109	52.16670	2.739115	993.5196	0.008534
**N-stage**	2.195298	1.048906	4.594628	0.033691	0.586906	0.104827	3.285962	0.544291
**Risk score**	1.156975	1.066262	1.255405	0.000465	1.094405	0.956241	1.252532	0.000147

Note: HR: Hazard Ratio.

### Analysis of the immune status of the high-risk and low-risk population

**Figure 8:**
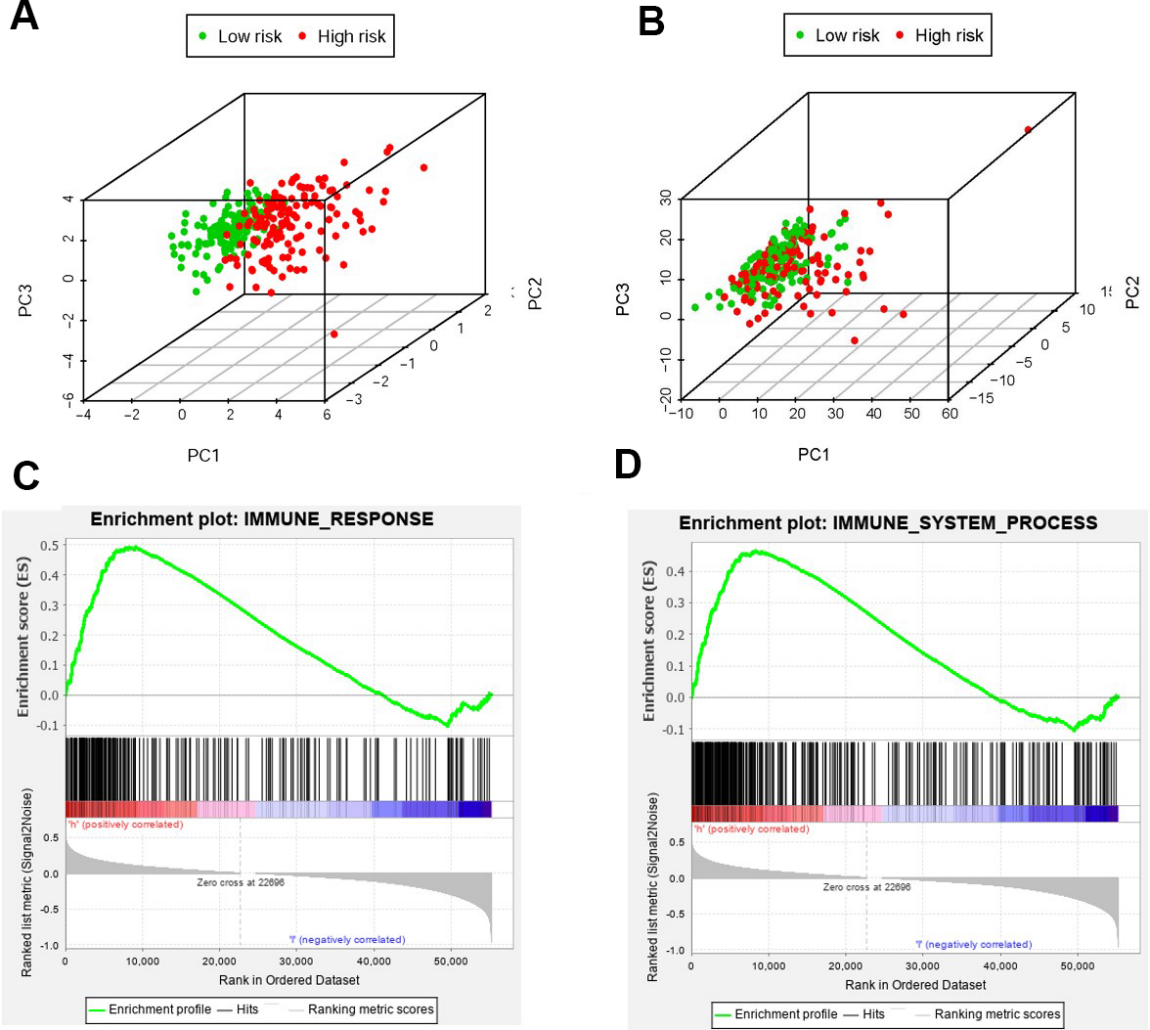
**The analysis of immune status of the high-risk and low-risk population by PCA and GSEA.** PCA of the high-risk group and the low-risk group was conducted based on the immune related gene sets (**A**). PCA of the high-risk group and the low-risk group was performed based on the whole protein-coding gene sets (**B**). GSEA implied remarkable enrichment of immune-related phenotype in the high-risk group (**C** and **D**). The Normalized Enrichment Score (NES) were 1.58 and 1.59 respectively.

We employed the PCA to detect the different distribution patterns between the low-risk group and the high-risk group by the immune gene sets and the genome-wide expression sets. In the IRGs set, the low-risk group and the high-risk group were observably separated with the lower immune scores in the low-risk group (Figure 8A). While we didn’t detect the significant the separation of the immune scores on the basis of the genome-wide expression profiles (Figure 8B). The results of GSEA further proved the functional annotation, with the more active immune-related responses and processes in the high-risk group (Figure 8C and 8D).

**Figure 9:**
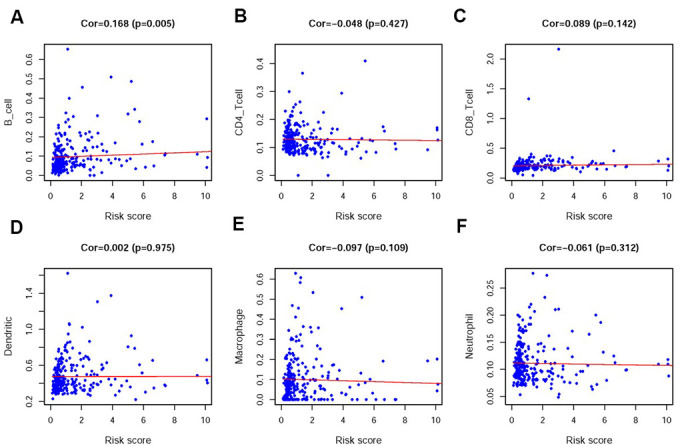
**Relationships between the IRRS and infiltration abundances of six types of immune cells.** The relationships were examined using PCA. B cells (**A**); CD4 T cells (**B**); CD8 T cells (**C**); dendritic cells (**D**); macrophages (**E**) and neutrophils (**F**).

In order to verify whether the immune genome accurately reflects the status of the tumor immune microenvironment, we analyzed the relationships between the sIRlncRs and immune cell infiltration (Figure 9A–9F). We found that only B cell showed the most significant relationship with sIRlncRs (Figure 9A).

### SNHG16 and ADAMTS9-AS1 were respectively high-expression and low-expression in pRCC patients especially in female with advanced T-stages

**Figure 10:**
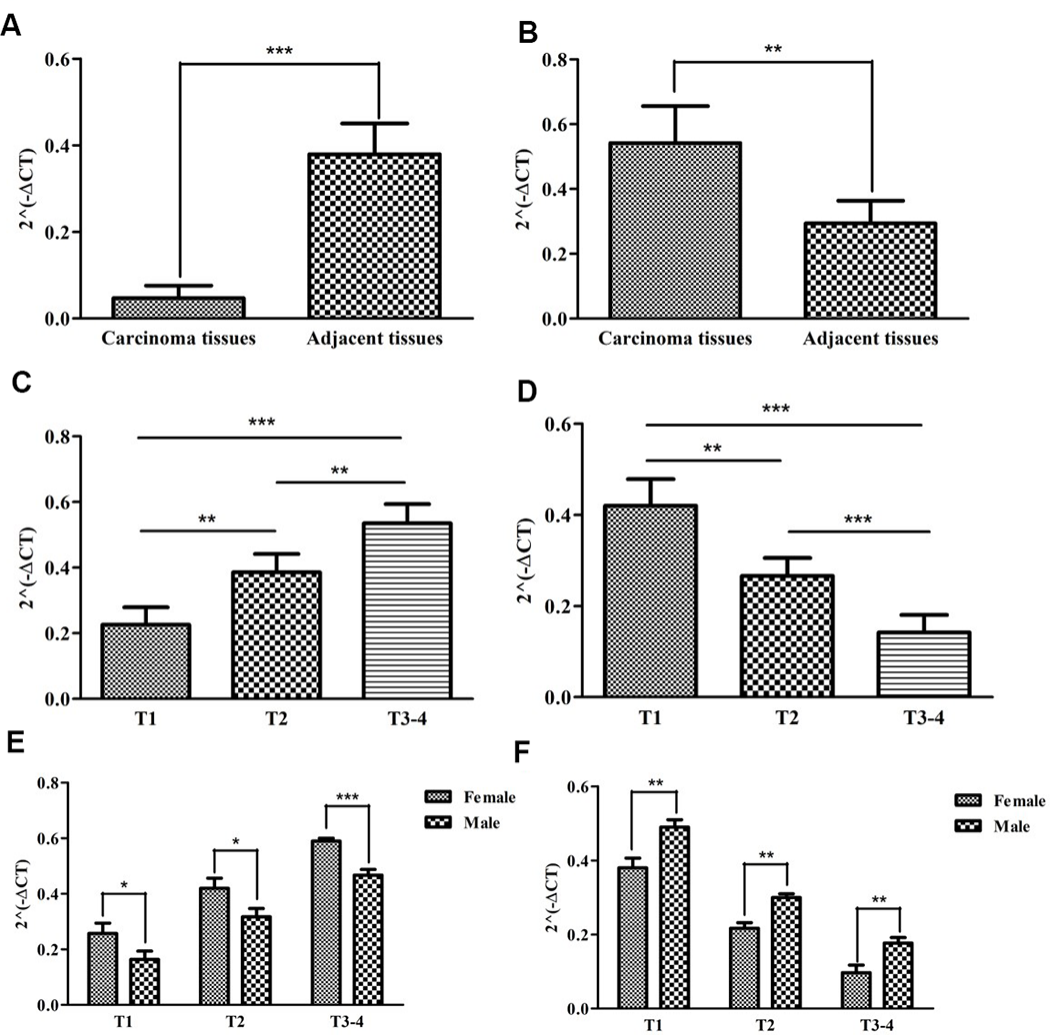
**The expression levels of two sIRlncRs and their correlations with gender and T-stages.** The results of RT-qPCR of the two sIRlncRs’ expression levels in pRCC tissues and adjacent tissues. The expression levels of SNHG16 in carcinoma tissues and adjacent tissues (**A**). The expression levels of ADAMTS9-AS1 in carcinoma tissues and adjacent tissues (**B**). The expression levels of SNHG16 in pRCC tissues with different T-stages (**C**). The expression levels of ADAMTS9-AS1 in pRCC tissues with different T-stages (**D**). The expression levels of SNHG16 in female and male patients (**E**). The expression levels of ADAMTS9-AS1 in female and male patients (**F**). The data are expressed as the means ± SD. ***P<0.001, **P<0.01, *P<0.05.

We previously explored the roles of SNHG16 and ADAMTS9-AS1 on predicting prognosis by bioinformatics methods (Supplementary Figure 1). To further verify the relationships between sIRlncRs and the clinicopathologic features, as well as discover the roles of sIRlncRs on indicating clinical prognosis, we detected the expression levels of SNHG16 and ADAMTS9-AS1 in carcinoma and adjacent tissues of pRCC patients with different genders and T-stages. Given age was insignificant associated with the five sIRlncRs, it was removed in the verification process. As illustrated in Figure 10, compared with adjacent tissues, the higher expression of SNHG16 (Figure 10A) was detected in carcinoma tissues, but ADAMTS9-AS1 (Figure 10B) showed the reversed expression level. Besides, the expression of SNHG16 (Figure 10C) and ADAMTS9-AS1 (Figure 10D) were significantly more and lower respectively in T3 and 4 stages compared with that in earlier T-stages. Female patients were detected to obtain the higher expression levels of SNHG16 (Figure 10E) and the lower expression levels of ADAMTS9-AS1 (Figure 10F) than male.

## DISCUSSION

The significance of tumor immunization activities on tumorigenesis and development and the individual variation at the genetic level attracted a growing body of researchers pay attention to discover the implications of differential genes which were potential for distinguishing pRCC patients responding heterogeneously and predicting prognosis, thus increasing miRNAs and lncRNAs are being identified to be labelled on the immune correlation [20–23] However, the mechanisms of IR-lncRs on pRCC at the level of whole genome have yet to be adequately elucidated [24].

To detect the roles of lncRNAs in assessing prognosis, Seema Khadirnaikar established a lncRNA immune prognostic signature score model based on seven lncRNAs, and demonstrated a negative correlation between the score and survival of patients with ccRCC [25]. To elucidate the functional roles and regulatory mechanism of lncRNAs in pRCC, Xin Z. constructed a pRCC-associated competing endogenous RNA network, with 57 lncRNAs, 11 miRNAs, and 28 mRNAs, besides, they identified three mRNAs (ERG, RRM2 and EGF) regarded as negative indicators of prognosis [26]. Ze G. also developed an assessing model based on the expression profile of five mRNAs (CCNB2, IGF2BP3, KIF18A, PTTG1, and BUB1) to predict the survival of pRCC patients [27]. These discoveries offer us inspiration to focus subsequent studies on detecting some specific IR-lncRs and developing a practical immune-related risk scoring model to assess the immune status and indicate prognosis of pRCC patients.

In the present study, 322 pRCC patients were enrolled in a genome-wide analysis for lncRNAs, combining with 311 IRGs screened in Molecular Signatures Database v4.0 (Immune system process M13664, Immune response M19817), and 17 IR-lncRs were identified eventually. We further detected the relation between the prognosis of pRCC patients and the expression levels of the 17 IR-lncRs, of which 5 sIRlncRs indicated the significant correlation with OS. Utilizing multivariate Cox and risk score model, we further identified 4 IR-lncRs to establish a risk evaluating model which was available to distinguish pRCC patients into the high-risk group and low-risk group with obviously differences of OS. Due to the molecular heterogeneity, the accuracy and sensitivity of the present clinical risk factors in predicting the survival of pRCC patients remain unsatisfying, we further validated the predicting value of the three sIRlncRs by multivariate analysis. We found the 4 sIRlncRs were independent of traditional risk factors and molecular characteristics. According to the IRGs set, the low-risk group and the high-risk group tended to be divided into two parts, with the low-risk group having lower immune scores than the high-risk group. GSEA was employed to further verify the functional annotation, and we found the more abundant immune-related responses and processes in the high-risk group. Therefore, immune-related scores are bound up with the immune status of pRCC, with higher scores indicating the poor prognosis. Besides, we found only B cell showed the most prominent relationship with sIRlncRs, which is probably due to the remarkable effects of macrophage polarization on the tumorigenesis, progression, prognosis and tumor behaviors of pRCC. The results motivated us to further discover the underlying functions and mechanisms in future studies.

Furthermore, SNHG16 and ADAMTS9-AS1 were further verified in 22 pRCC samples, and the significant correlations between the two sIRlncRs and T stages and genders were detected. We found female patients obtained the higher expression levels of SNHG16 and the lower expression levels of ADAMTS9-AS1 than male. It is probably due to the transcription factors which affect genes expression levels are different between male and female. Some sex-biased genes have transcription factors with different expression levels between different genders in the promoter region where genes can be activated. Besides, sex chromosomes and hormones control the differences between male and female, and these differences could eventually affect transcription factors [28]. These findings suggest that the risk evaluating scores based on the 4 sIRlncRs can contribute to identify the high-risk patients from patients with the same clinical or molecular characteristics, thereby realize individualized and appropriate therapeutic strategy.

In the analyzing process, the large numbers of pRCC patients were enrolled to further enhance the reliability and persuasion in guiding clinical strategic decisions. Besides, some specific IR-lncRs with remarkable differences under variable risk factors have been further corroborated to be implicated in the progression and prognosis of pRCC. More importantly, these IR-lncRs as the molecular bioindicators, showed significant potency in forecasting and evaluating the OS.

Although we elucidated the roles of sIRlncRs on forecasting prognosis and verified the expression levels of SNHG16 and ADAMTS9-AS in tumor tissues, some limitations remain to be further discussed. Firstly, we didn’t combine with the detection of proteomics, metabolomics and immunogenomics. Then, the practical application values of these sIRlncRs have yet to be adequately elucidated and need to be further wide verification. Thirdly, except for SNHG16 and ADAMTS9-AS, other sIRlncRs included in the IRRS model are also needed to be explored.

In conclusion, we comprehensively analyzed and verified the effects of IR-lncRs on forecasting clinical prognosis of pRCC. The results will contribute to develop a reliable and referable risk evaluating model and provide new insight into the immune-related researches and treatment strategies.

## MATERIALS AND METHODS

### Ethics statement

Informed consent forms have been signed by all patients before this study. The research protocol has been approved by the Ethics Committee of The First Affiliated Hospital of Chongqing Medical University and is based on the ethical principles of medical research involving human subjects in the Helsinki Declaration.

### Clinical renal samples

A total of 22 clinical samples of pRCC and corresponding adjacent tissues were collected from patients who received treatment in the First Affiliated Hospital of Chongqing Medical University from October 2018 to December 2019. The collected tissue samples were immediately frozen in liquid nitrogen and then stored at –80°C until RNA extraction.

### Download and pretreatment of data

Transcriptome RNA-sequencing of pRCC samples were downloaded from the The Cancer Genome Atlas (TCGA) data portal (https://portal.gdc.cancer.gov/), that contained data from 32 non-tumor tissues and 289 pRCC tissues. The clinical data about these patients was also downloaded and extracted (the OS of patients≤30 days were excluded because these patients probably died for unpredictable factors such as infection and hemorrhage). Raw data was collected to do further analyses. These data were currently updated in November 11, 2019. RNA-seq results were combined into a matrix file by a merge script in the Perl language (http://www.perl.org/). The Ensembl IDs of genes was converted into a matrix of gene symbols by the Ensembl database (http://asia.ensembl.org/index.html).

### Immune-related long non-coding RNA acquisition

The Molecular Signatures Database v4.0 (Immune system process M13664, Immune response M19817, http://www.broadinstitute.org/gsea/msigdb/index.jsp) was used to explore the immune-related gene participating in the immune process. Immune related gene was used to establish the immune score of pRCC gene by GSEA. The correlation between the immune score and the expression of lncRNA in pRCC patients was analyzed by Pearson correlation analysis. IR-lncRs was identified by a criterion of |r|>0.8 and P<0.001.

### Survival-related IR-lncRs

IR-lncRs associated with clinical outcomes in pRCC patients were regarded as survival-related IR-lncRs (sIRlncRs). Univariate COX analysis was used to screen sIRlncRs (p<0.01). Hazard ratio (HR) was used to specified sIRlncRs into protective and deleterious portion. These sIRlncRs were selected for follow-up study.

### Establish the immune-related risk score model (IRRS)

To verify the reliability, sIRlncRs were submitted for the multivariate analysis, while the integrated sIRlncRs were still used as an independent prognostic indicator to develop the IRRS (p<0.05) (Table 3). In order to explore the heterogeneous clinical prognostic outcomes, based on the differential expression of sIRlncRs, we performed a risk score model to divide pRCC patients into the high-risk group and the low-risk group according to the median risk score which was chosen as the cutoff point. IRRS was established based on the expression data multiplied by Cox regression coefficients. The formula was as followed, [Expression level of AP001267.3 * (-1.213353)] + [Expression level of SNHG16 * (0.581303)] + [Expression level of AC026471.3* (0.779272)] + [Expression level of ADAMTS9-AS1* (-0.233562)]. The values of IRRS were employed to evaluate various subtypes of pRCC patients. To further investigate the relevance of the sIRlncRs and clinicopathological features of pRCC, we analyzed the relationship between the IRRS and clinicopathologic characteristics, of which the “TNM staging method” is the most common way to describe the tumor status. The division of “T-stage” pRCC was based on the maximum diameter of tumors and extent of tumor invasion, with the bigger tumors and more extensive invasive statues in the more advanced T stages. “N-stage” reflects the lymph node metastasis conditions with more metastatic lymph nodes in the more advanced N stages. “M-stage” is distinguished according to whether the tumor exhibits distant metastasis, and advanced M stages usually represent poor tumor conditions. In addition, “stage” is a comprehensive method combining T-stage, N-stage and M-stage to divide patients with pRCC into I, II, III and IV stage.

**Table 3:** The results of multivariate Cox regression coefficients.

**Gene**	**Coefficients**	**HR**	**HR 95% low**	**HR 95% high**	***P* value**
**AP001267.3**	-1.213353	0.297199	0.064159	1.376692	0.012084
**SNHG16**	0.581303	1.788367	1.069646	2.990014	0.026643
**AC026471.3**	0.779272	2.179885	1.271855	3.736197	0.004586
**ADAMTS9-AS1**	-0.233562	0.791708	0.645718	0.970705	0.024713
**AC021054.1**	-2.312864	0.098977	0.051808	0.189094	0.187025

Note: HR: Hazard Ratio.

### Bioinformatics analysis

The survival ROC curve was employed to verify the prognostic performance through the survival ROC package of the R software. The survival time, survival status and risk scores of patients with pRCC were used to predict the prognosis over a 5-year period, then the ROC curve was drawn and the value of AUC was calculated. Abscissa was the false positive rate and ordinate represented the true positive rate. Principal component analysis (PCA) was displayed to demonstrate the expression levels of pRCC samples and gene set enrichment analysis (GSEA) was used to detect the different functional phenotypes between the low-risk group and high-risk group. The tumor infiltrating immunocytes were evaluated through the TIMMER database. The levels of immune infiltration in pRCC patients were downloaded, and the relationship between IRRS and immune cell infiltration was calculated.

### Real-time quantitative PCR

Triazole (Invitrogen) was used to extract total RNA from tissues and cell lines under various experimental conditions according to the manufacturer's instructions. cDNA Synthesis Kit (Osaka, Japan of TaKaRa) combining with RNA (1μg) was utilized to reverse transcribed cDNA. The reaction steps were as follows: 37°C for 15min, 85°C for 5s, and quantitative polymerase chain reaction (qPCR) was performed on an ABI 7500 real-time PCR system (Applied Biosystems) using SYBR-Green method (TaKaRa). The reaction cycle conditions were performed (95°C 30s, followed by 40 cycles of 95°C for 5 s and 60°C for 34 s). Relative expression level of lncRNAs normalized to β-actin was calculated by the 2^−ΔCt^ method. The primer sequences are shown in Table 4. Three replicate assays were performed for each cDNA sample.

**Table 4:** The primer sequences of ADAMTS9-AS1 and SNHG16.

**ADAMTS9-AS1**	F primer (5’-3’)	ATAACACTCCTAACCCTGCTCC
	R primer (5’-3’)	CTGATCCTGCCTTCTGATGCT
**SNHG16**	F primer (5’-3’)	GGACCCAAAGTGCCATGTCT
	R primer (5’-3’)	ATGAAGCCCAAAGAACGCAT
**β-actin**	F primer (5’-3’)	AAACGTGCTGCTGACCGAG
	R primer (5’-3’)	TAGCACAGCCTGGATAGCAAC

Note: F primer: forward primer; R primer: reverse primer.

### Statistical analysis

Univariate Cox regression analysis and Pearson correlation analysis were used to identify the target IR-lncRs. Kaplan-Meier curve was used to evaluate the OS between low-risk group and high-risk group. Univariate and multivariate Cox regression analysis were used to verify the independent prognostic factors for pRCC patients. All statistical analysis was conducted using SPSS21.0 software (SPSS Inc, Chicago, IL) and GraphPad Prism5 (GraphPad Software Inc, La Jolla, CA). Varieties in clinical parameters were determined using independent t-tests. *P*<0.05 was considered significantly statistical difference.

## Supplementary Material

Supplementary Figure 1
