# Supplementary material for: Bioinformatics profiling integrating a four immune-related long non-coding RNAs signature as a prognostic model for papillary renal cell carcinoma
Source: Aging (Albany NY). 2020 Jul 27;12(15):15359–73. doi: 10.18632/aging.103580 (PMC7467365; doi:10.18632/aging.103580)
Supplement: Supplementary Figure 1 [file aging-12-103580-s001..pdf]

SUPPLEMENTARY FIGURE

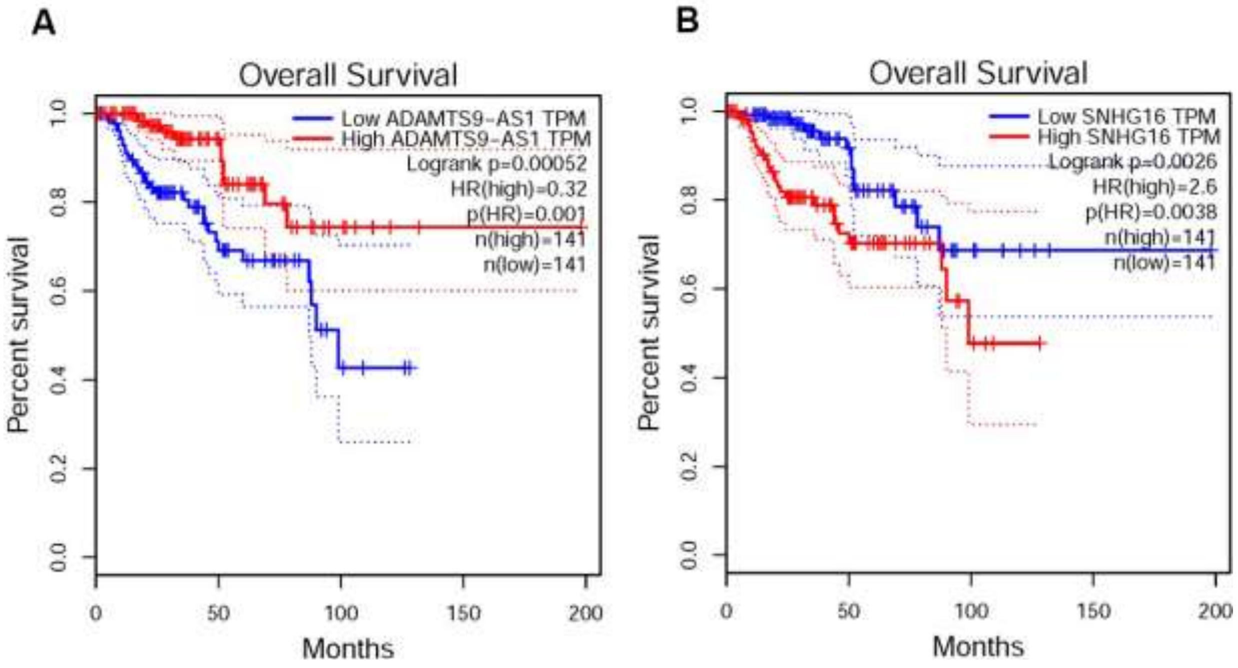

Supplementary Figure 1. The survival curve of ADAMTS9-AS1 (**A**) and SNHG16 (**B**) in pRCC patients.
